# CRISPR Manipulation of Age-Related Macular Degeneration Haplotypes in the Complement System: Potential Future Therapeutic Applications/Avenues

**DOI:** 10.3390/ijms25031697

**Published:** 2024-01-30

**Authors:** Ahmed Salman, Michelle E. McClements, Robert E. MacLaren

**Affiliations:** 1 Nuffield Laboratory of Ophthalmology, Nuffield Department of Clinical Neurosciences, University of Oxford, Oxford OX3 9DU, UK; 2 Oxford Eye Hospital, Oxford OX3 9DU, UK

**Keywords:** CRISPR, age-related macular degeneration (AMD), single-nucleotide polymorphisms (SNPs), retina, gene therapy

## Abstract

Age-related macular degeneration (AMD) is the leading cause of irreversible vision loss among the elderly in the developed world. Whilst AMD is a multifactorial disease, the involvement of the complement system in its pathology is well documented, with single-nucleotide polymorphisms (SNPs) in different complement genes representing an increased risk factor. With several complement inhibitors explored in clinical trials showing limited success, patients with AMD are still without a reliable treatment option. This indicates that there is still a gap of knowledge in the functional implications and manipulation of the complement system in AMD, hindering the progress towards translational treatments. Since the discovery of the CRISPR/Cas system and its development into a powerful genome engineering tool, the field of molecular biology has been revolutionised. Genetic variants in the complement system have long been associated with an increased risk of AMD, and a variety of haplotypes have been identified to be predisposing/protective, with variation in complement genes believed to be the trigger for dysregulation of the cascade leading to inflammation. AMD-haplotypes (SNPs) alter specific aspects of the activation and regulation of the complement cascade, providing valuable insights into the pathogenic mechanisms of AMD with important diagnostic and therapeutic implications. The effect of targeting these AMD-related SNPs on the regulation of the complement cascade has been poorly explored, and the CRISPR/Cas system provides an ideal tool with which to explore this avenue. Current research concentrates on the association events of specific AMD-related SNPs in complement genes without looking into the effect of targeting these SNPs and therefore influencing the complement system in AMD pathogenesis. This review will explore the current understanding of manipulating the complement system in AMD pathogenesis utilising the genomic manipulation powers of the CRISPR/Cas systems. A number of AMD-related SNPs in different complement factor genes will be explored, with a particular emphasis on factor H (C*FH*), factor B (C*FB*), and complement C3 (*C3*).

## 1. Introduction

Several reasons make the retina an exceptional model for developing gene therapy treatments, with dozens of adeno-associated virus (AAV)-based clinical trials already established in the last two decades. The retina is easy to access surgically, it has a relatively immune-privileged blood–retinal barrier, and there are several well-established tests to evaluate retinal structure and function after therapy. The retina is the first organ where CRISPR editing was implemented, in The BRILLIANCE trial (EDIT-101), which aimed to edit the *CEP290* gene to treat Leber congenital amaurosis as the first in-human CRISPR gene therapy trial [1,2], which shows the advantages the retina offers as a gene therapy target. The involvement of genetic variants in the complement system in AMD has generated interest in potential gene therapy treatments [3]. Drusen accumulation at the site of inflammation is a hallmark of AMD which deters the retinal pigment epithelium’s (RPE) ability to maintain support of the photoreceptors [4]. In addition, chronic inflammation involving dysregulation of the complement system has long been implicated in the pathogenesis of AMD [5]. Numerous AMD-associated SNPs in the complement system have been identified in the literature across 33 discrete loci [3], with SNPs in *CFH*, *CFB*, and *C3* representing a large majority. The following sections will discuss the implications of using CRISPR manipulation of AMD risk/protective variants in the complement system as a potential therapeutic treatment, starting with the nature of the complement cascade amplification, expanding to targeting strategies for CRISPR editing of selected AMD variants, and ending with prospective implications of using such strategies for the treatment of AMD.

## 2. Brief Insight into the Amplification Nature of the Complement Cascade

**Figure 1:**
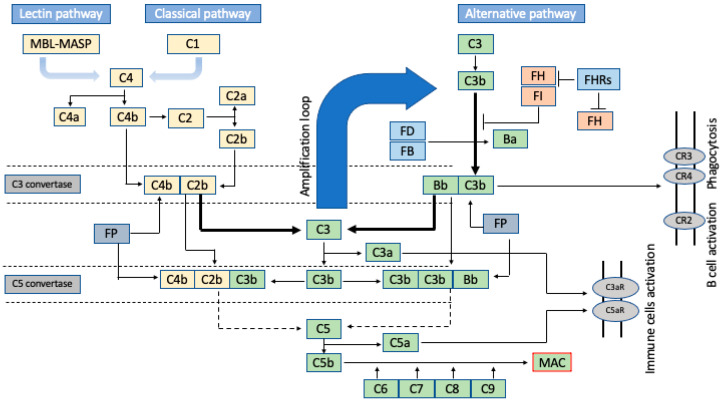
Schematic flow diagram of the complement system activation. The complement cascade initiated by three main pathways: the lectin pathway, the classical pathway (both in yellow), and the alternative pathway (indicated in green). All pathways converge in the formation of C3 convertase and C5 convertase (grey). Activation in the lectin and classical pathways starts with recruitment of C1 complex to cleave C4 into C4a and C4b. This also leads to cleavage of C2 into C2a and C2b. C3 convertase forms by binding C4b and C2b (dotted lines). The alternative pathway activation (indicated in green, where most AMD-related variants come from) starts with the hydrolysis of circulating C3 into C3b. C3 convertase forms through the binding of C3b to Bb, which forms from the cleavage of resident FB. C3 convertase continuously cleaves C3 into C3b; this cleavage makes C3b covalently bind to surfaces and blocks C3 convertase, resulting in the continuous cleavage of C3 into C3b and contributes to an amplification loop of complement activation (thick arrows). C3b also contributes to the formation of C5 convertase, being responsible for the cleavage of C5 into C5b and the subsequent recruitment of C6, C7, C8, and C9 to form the membrane attack complex (MAC), which creates pores on surfaces of pathogens, leading to lysis. The complement system is tightly regulated by negative (orange) and positive (light blue) regulators. FI and its cofactor, FH, promote inactivation of C3b. FH also promotes inactivation of C3 convertase. FHRs (factor H-related proteins) compete with FH for the binding of C3b and inhibits cleavage of C3b by FI. Properdin (FP), a plasma glycoprotein, is involved in the activation of the complement system (reviewed in ref. [12]). C3a and C5a (generated by cleavage of C3 and C5, respectively) bind to membrane-bound receptors C3aR/C5aR (grey ovals) to promote inflammation. C3b breakdown fragments bind membrane-bound receptors, CR3/CR4, and promote phagocytosis and B-cell activation. Bold arrows indicate direct interactions and dashed arrows indicate indirect interactions.

The complement system involves over 40 different proteins that are a critical part of the innate immune response. It is responsible for recognising and eliminating pathogens, cell debris, and immune complexes prior to the modulation of the (pathogen-specific) adaptive immune response ([6] and reviewed in [7]). The complement system discriminates between self and foreign antigens, marking the latter for elimination via phagocytosis or initiating the (tissue-damaging) inflammatory response. The complement system is activated by three different pathways (classical, lectin, and adaptive), all of which lead to enzyme activation, protein cleavage, and protein conformational changes that result in an immune response [5,8] (Figure 1). The classical and lectin pathways are quite similar, being are activated when the C1 complex binds to the Fc domain of antibodies in immune complexes or immunoglobulin activators [9]. This binding leads to the cleavage of the downstream complex C4 into C4a and C4b. C1 binding also leads to the cleavage of another complex, C2, into C2a and C2b. This leads to the formation of the C3 convertase, which splits C3 into C3a and C3b as part of the opsonic ingestion process of pathogens. C3b is the active component that leads to the formation of the classical pathway C5 convertase, which cleaves C5 into C5a and C5b, the latter of which initiates the formation of the pore-forming complex (C5b, C6, C7, C8, and C9). This leads to the formation of the lytic membrane attach complex (MAC) as part of a global inflammatory response [10,11].

The classical and lectin pathways target C3b deposition at antigen sites. In contrast, the alternative pathway, through spontaneous or protease-mediated C3 activation, leads to non-specific C3b complex deposits on surrounding surfaces [10,11]. It starts with the activation of C3 that is eventually cleaved to C3b by C3 convertase. The cleavage process starts with the binding of factor B (FB) to C3b to form the C3b-FB complex, which is then activated by factor D (FD) to form the protease complex C3 convertase (C3bBb), which cleaves C3 into C3b. The convertase-generated C3b can form more C3 convertase, allowing the alternative pathway to amplify exponentially [11]. As a result, higher amounts of C3b accumulate, driving the activation of C5 and the formation of the MAC. This amplification nature of the alternative pathway is advantageous in terms of providing a rapid and effective immune response but also dangerous as it requires stringent regulation to avoid host cell damage. The complement system is tightly controlled in an equilibrium, and deviation from that equilibrium is associated with disease (see ref. [11] for more details).

Whilst AMD is a multifactorial disease, the complement system has been identified as one of the main pathways in disease pathogenesis. A strong link between genetic variations in the complement system and AMD was demonstrated by genetic association. Genome-wide association studies (GWASs) using exon chips detected 52 (45 common and 7 rare) variants that are independently associated with AMD [3]. More than a third (19/52) of these variants reside in or near a gene of the complement system.

Complement factor H (CFH) and factor I (FI) are key inhibitors of complement activation. Genetic variants of these factors represent a risk factor for AMD due to their involvement in C3b cleavage and activation of the downstream complement cascade. CFH is the main inhibitor of the alternative pathway; it inhibits interactions of FB with C3b, obstructs the cleavage of C3 into C3a and C3b, and removes C3b from the C3aC3b complex, which downregulates the alternative pathway activity when activated excessively [13].

In addition to genetic variations in the *CFH* locus, variants in factor B (*FB*) and complement component 3 (*C3*) are among many other variants in the complement cascade that have also been associated with AMD [3,14,15]. Due to the complications that result from a dysregulated complement system with its many components in the pathogenesis of AMD, which is not the focus of this review, we will concentrate on introducing selected SNPs in *CFH*, *CFB*, and *C3* as examples for CRISPR manipulation of SNPs in these genes as a therapeutic strategy for AMD treatment.

## 3. The CFH-CFHR Haplotypes

**Figure 2:**
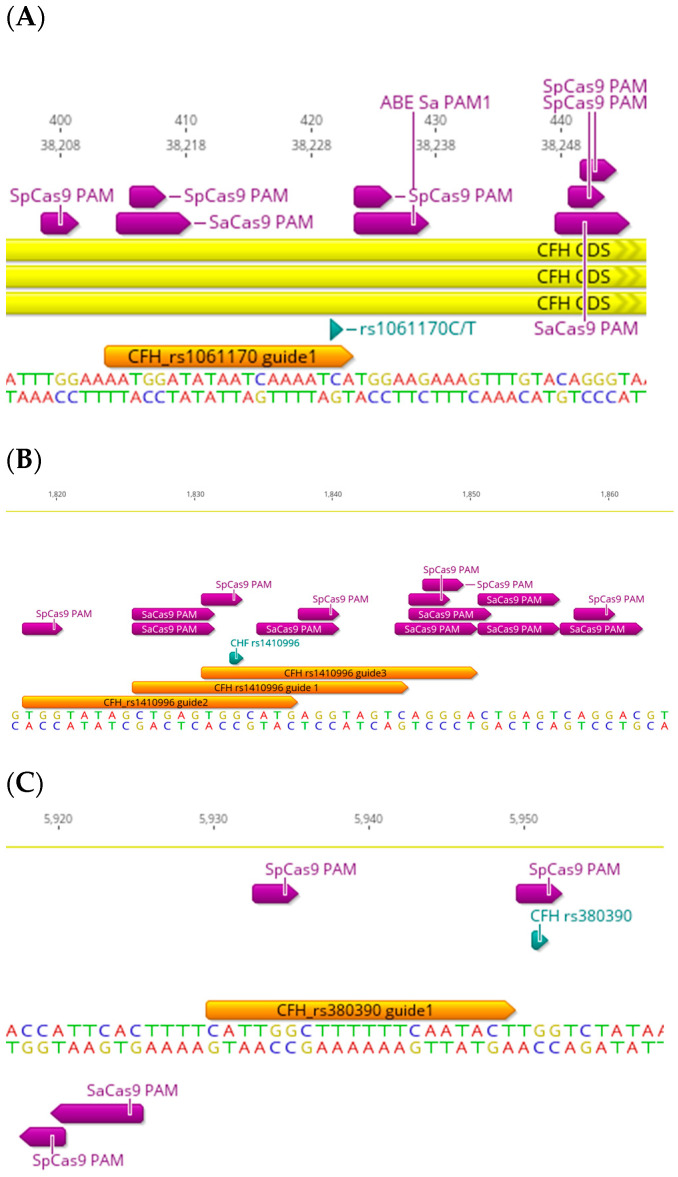
Targeting design of different AMD-related SNPs in *CFH* gene. Representative sequencing chromatograms in the *CFH* gene showing the locations of SNPs with varying AMD risk (green): rs1061170 (**A**), rs1410996 (**B**), and rs380390 (**C**). PAM sites of different Cas9 strains, *Streptococcus pyogenes* Cas9 (SpCas9), *Staphylococcus aureus* (SaCas9), and occasionally adenine base editor (ABE SaCas9) (purple arrows) and corresponding single-guide RNAs (gRNAs) (orange arrows) showing variety in the targeting approach for targeting the SNPs.

The *CFH* locus encodes FH and its smaller splice variant, FH-like 1 protein, as well as the very similar FH-related 1-5 (FHR-1-5) proteins [16,17]. These proteins show high homology to the surface recognition domains of FH, but their function is not yet completely understood. Recent findings indicate they compete with FH and FHL-1 for fine-tuned complement activation [18]. The strongest AMD genetic association in the complement system identified to date was reported in the common p. Tyr402His (rs1061170) variant in the complement factor H (*CFH*) gene [19,20,21,22], which is a missense coding SNP (C>T) and the most common AMD-related variant reported in the literature with the following allele frequencies: C = 0.32133 and T = 0.67867. Possession of at least one histidine at amino acid position 402 of the *CFH* gene increases the risk of AMD 2–7-fold and may account for approximately 50% of the attributable risk of AMD. *Streptococcus pyogenes* Cas9 (SpCas9) has been harnessed as the most widely used Cas9 variant for genome engineering, including gene disruption, epigenetic modulation (activation/repression), and single base pair conversion in various organisms and cell types [23,24,25,26,27,28]. rs1061170 is targetable by SpCas9 since there are multiple protospacer adjacent motif (PAM) sites, short stretches of nucleotides required for target recognition (NGG for SpCas9) [29,30], within a reasonable proximity of the SNP (Figure 2A). The closest PAM site is only one base pair away in the 5′ direction (downstream) of the SNP on the forward strand, which is ideal since the seed sequence in the gRNA (first 10–12 bp proximal to the PAM site) binds first to the Cas9-gRNA complex when it starts annealing to the target DNA [31,32]. In theory, rs1061170 is also targetable by *Staphylococcus aureus* (SaCas9) (Figure 2A), another key effector of type II CRISPR proteins that is widely used due to its adaptability and small size, meaning it can be packaged into a single adeno-associated virus (AAV) [33]. There is a PAM site (NNGRRT for SaCas9); however, the closest PAM site is 18 nucleotides away in the 5′ direction of the SNP on the forward strand, which might make it hard to target since the SNP falls outside the seed sequence. On the other hand, rs1061170 is not an ideal target for single-nucleotide base editing (BE). Although it is a C>T transition, which is amendable for correction by single-base editing with cytidine base editors (CBEs, the other BE is the adenine base editor (ABE)), and despite the presence of adjacent PAM sites (the non-classical TGGAAG for SaCas9 (the classical is NNGRRT) and NGG for SpCas9 CBEs) on the forward strand, however, the pathogenic “T” nucleotide is only two base pairs away from the PAM sites in the 5′ direction. Therefore, it will unlikely be edited since the editing window for SaCas9 CBEs is 3–14 base pairs (away from the PAM site) and 4–8 base pairs for SpCas9 CBEs. In both scenarios, the editing window is not optimal (Figure 2A). The second closest PAM site (for CBE SaCas9) is 17 bp away in the 5′ direction, which falls outside the optimal seed window.

There are several common coding and non-coding AMD-related SNPs in the *CFH* gene (Table 1) which are also targetable by CRISPR. Some common non-coding SNPs represent a good target for CRISPR editing, such as rs1410996 and rs380390, both of which are intron pathogenic variants (rs1410996 is an AMD risk factor too) [22,34] (Table 1). rs1410996 is a G>A transition (allele frequencies: G = 0.054453 and A = 0.45547). CRISPR targeting can be guide-dependant since the efficiency of gRNAs can vary due to many factors, such as gRNA sequence, length, and its secondary structure, as well as the availability and proximity of a suitable PAM site [35]. rs1410996 is targetable by SpCas9, SaCas9, and another Cas variant, xCas9 (with the “NG” PAM site) (Figure 2B). There are more available SpCas9 PAM sites (two) which allow design flexibility of the gRNA, unlike SaCas9, where only one PAM site is available within the targeting range (20 bp). rs1410996 is also a candidate for base editing with ABEs since it is a G>A SNP. There are multiple PAM sites for both SaCas9 and SpCas9 ABEs, the most suitable is with the target “G”, being 12 and 8 base pairs proximal to the PAM site for the SaCas9 and SpCas9 ABEs, respectively (for a base editing window of 3–14 bp for SaCas9 and 4–8 bp for SpCas9) (Figure 2B). As for rs389390, it is a G>C transversion (also reported to be a G>A transition and G>T transversion, NCBI reference SNP report), with allele frequencies of G = 0.3586, T = 0.0001, and C = 0.6413. It is only targetable by SpCas9, with one available PAM site where the SNP “G” is part of the PAM site (Figure 2C); it is also not a candidate for base editing since it is a G>C transversion.

## 4. The CFB Haplotypes

**Figure 3:**
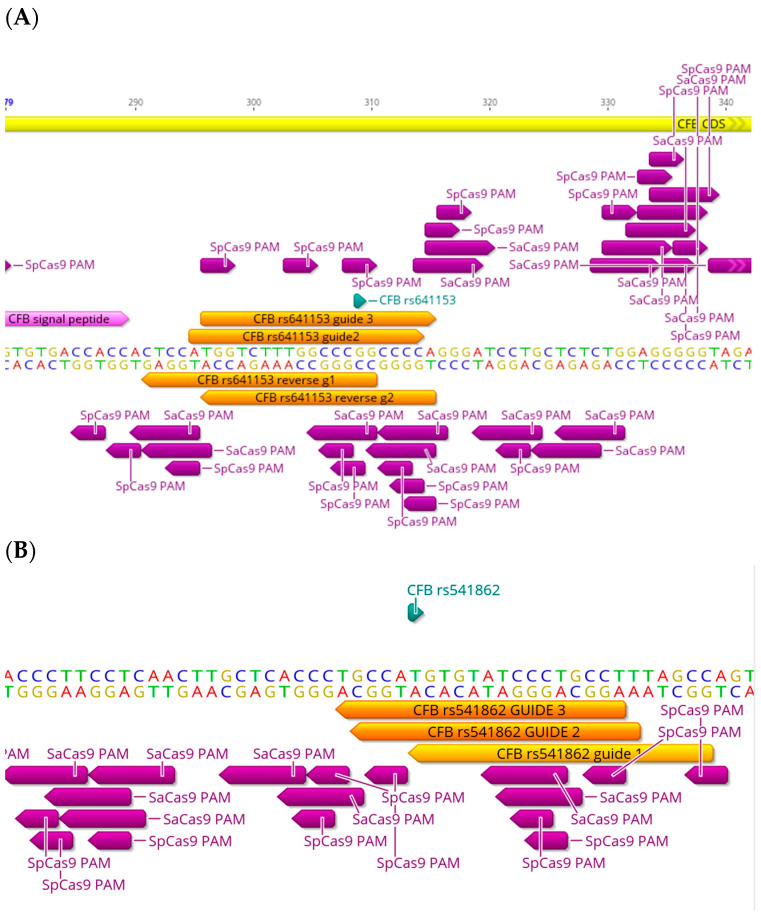
Targeting design of different AMD-related SNPs in *CFB* gene. Representative sequencing chromatograms in the *CFB* gene showing the locations of different AMD-related SNPs (green): rs641153 (**A**) and rs541862 (**B**). Locations of PAM sites of the Cas9 strains SpCas9 and SaCas9 are shown (purple arrows), as well as corresponding gRNAs (orange arrows) showing variety in targeting approach for targeting the SNPs.

CFB is mainly produced by hepatocytes and is expressed in the choroidal plexus in humans (EMBL-EBI database) and, at low levels, in the retinal pigment epithelium (RPE) [36]. Numerous studies show that genetic variants in CFB can be protective against AMD [37,38,39], whereas other studies show a predisposing role [3]. CFB, interacting with C3b through its Bb subunit, is a positive regulator in the complement cascade. This interaction facilitates the formation of C3 convertase and the MAC (refer to Section 1 for more details). CFB has been shown to be associated with a higher risk of developing AMD; however, studies have also reported variants such as R32Q (rs641153) and L9H (rs4151667) as being protective against AMD [3,37]. rs641153 is a G>A transition coding SNPs (allele frequencies: G = 0.90424, A = 0.09575) that can be targeted with SpCas9, SaCas9, xCas9 (both “NG” and “GAT” PAMs), and Nme2Cas9 (with the more specific NNNNCC PAM site) (Table 1). The location of this SNP in coding exon 1 of the *CFB* gene allows flexibility in the PAM site choice. For example, there are four SpCas9 PAM sites (two on the forward strand and two on the reverse strand), all within a targetable range (Figure 3A). Since rs641153 is a G>A transition SNP, it is amenable to base editing with ABEs. For example, among several SpCas9 and SaCas9 PAM sites, there are two SpCas9 PAM sites on the forward strand, where the target SNP is six and seven base pairs proximal to the PAM site. There are also two SaCas9 PAM sites on the forward strand, where the target SNP is five and six base pairs proximal to the PAM site, placing them in what are considered to be optimal locations for base editing. Another two SaCas9 PAM sites are present on the reverse strand, where the target SNP is 13 and 14 bp proximal to the PAM site; however, these fall at the end of the seed window, and editing will likely be less efficient (Figure 3A).

rs541862 is a T>C transition intronic variant which has been shown to have a significant association with AMD risk (allele frequencies: T = 0.87890 and C = 0.12110) [39]. There are three SpCas9 PAM sites on the reverse strand, where the target SNP is within a targetable range (gRNAs shown in Figure 3B). There are also two SaCas9 PAM sites adjacent to the target SNP on the reverse strand, where the SNP is four and eight base pairs in the 5′ end of the gRNA. These are more suitable for base editing with ABEs than active SaCas9 due to the distance of the SNP from the PAM site. As shown in the above examples, the strategy for CRISPR targeting depends on the availability, and proximity, of the PAM sites adjacent to the SNP, which can determine the Cas9 variant and the editing strategy, such as random indels using SpCas9/SaCas9 or the more specific single-base editing approach using ABEs/CBEs. All genome-editing strategies require consideration of off-target events.

## 5. The C3 and Other Functional Haplotypes

**Figure 4:**
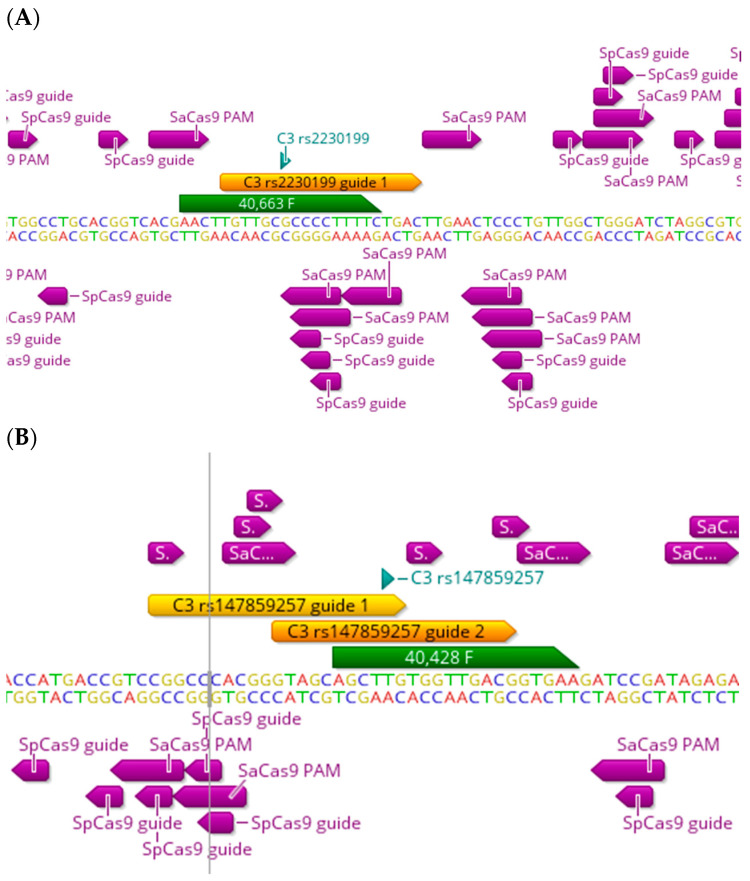
Targeting design of different AMD-related SNPs in *C3* gene. Representative sequencing chromatograms in the *C3* gene showing the locations of different AMD-related SNPs (green): rs2230199 (**A**) and rs147859257 (**B**). Locations of PAM sites of the Cas9 strains SpCas9 and SaCas9 are shown (purple arrows), as well as corresponding gRNAs (orange arrows) showing variety in the targeting approach for targeting the SNP.

There are several functional variants in almost all complement genes which are associated with an increased risk of AMD, with some showing a protective association too [3]. Complement component 3 (C3) plays a central role in the activation of the lectin, classical and alternative pathways (for a recent review, see [5]), and the C3 convertase, which cleaves C3 into C3a and C3b as a central step in the activation of the complement cascade. rs2230199 and rs147859257 are pathogenic missense variants in coding exons 10 and 11, respectively, in the *C3* gene, and have been reported to be associated with an increased risk of AMD [3,14,40,41,42]. rs2230199 is a C>G transversion (allele frequencies: G = 0.84853 and C = 0.15147) which is amenable to editing by SaCas9 and Nme2Cas9 (Table 1). There is one PAM site for SaCas9 available; however, the target “C” SNP lies at position 13, away from the PAM site (Figure 4A), which makes targeting it a little challenging for both active SaCas9 and the ABE SaCas9, since editing is likely to occur closer to the PAM site. As for rs147859257, it is a T>G transversion (with allele frequencies: T = 0.9950 and G = 0.00493), and there are two PAM sites available, one for both SaCas9 and SpCas9 (Figure 4B). The target “G” SNP lies within a more reasonable range for active Cas9 editing, between positions 11 and 2. This SNP is not amenable to base editing.

On the other hand, complement factor I (factor I (FI)), which is encoded by the complement factor I (*CFI*) gene, is a normal plasma protein whose function is to downregulate the alternative complement pathway by the cleavage of C3b into inactive iC3b [43]. The importance of FI as a regulatory complement protein has been reinforced by reports that a collection of complement SNPs increasing the risk for AMD, which is known as the “complotype”, alters the downregulation feedback cycle of C3b by FI [44], highlighting the complex nature of the complement system in AMD. There are conflicting reports on the association of CFI variants such as rs141853578 and rs10033900 with AMD (Table 1), where some reports show an increased risk of AMD [45,46], some show a protective effect [45,46,47,48], and others show no association [49,50].

## 6. Implications of Future CRISPR Therapeutics for AMD-Related SNPs

The genetic association of AMD can be classified into two categories: common variants with a generally limited effect size (except the common p.Tyr402His in FH) and rare variants, which can have a large effect on the AMD pathology. While rare variants with a significantly large effect on the development of AMD have been reported [3], for many of those variants, a significant association with AMD could not be established since they are too rare to trigger an association with large database settings [51]. For that reason, a combination of genetic association and protein analyses are required to establish their clinical significance.

Numerous clinical trials on the genetic associations between the complement system and AMD (reviewed in [52]) have shown limited success, which emphasises the need for a deeper understanding of the molecular mechanisms of how the complement system contributes to the development of AMD. In the following paragraphs, we will present an overview of the implications of using CRISPR in manipulating disease variants in AMD, giving examples of several AMD-related SNPs in the complement system and the potential effect of manipulating them with CRISPR.

CRISPR targeting of pathogenic variants with active Cas9 appeals as a promising therapeutic approach since specific knockout of pathogenic AMD SNPs could potentially result in a non-pathogenic variant. For example, a study by Hecker et al. looked at a risk variant of AMD in the *C3* locus (rs2230199) and showed a trend towards elevated levels of the complement activation marker C3a [53]. rs2230199 is a C>G transversion; if specific knockdown of the pathogenic (G) allele by random indels (generated by active Cas9 targeting) is possible, then indeed this could be an ideal strategy. However, achieving allele specificity with active Cas9 can be challenging.

In the case of protective SNPs, a knockout of the wild-type variant into the protective variant seems to be logical. For example, the same study by Hecker et al. looked at the plasma levels of AMD patients carrying a protective haplotype in the *C2/CFB* locus (rs4151667), which has been found to be associated with reduced levels of the complement activator Ba, whereas another protective haplotype in the *CFB* locus (rs641153) was found to be associated with decreased levels of another complement activator (C3a) [53]. This tendency towards reduced complement activation markers with protective haplotypes in the *CFB* locus could potentially be a target for CRISPR manipulation. For rs4511667, a T>A transversion intron variant (allele frequencies: T = 0.96091 and A = 0.03909), specific knockdown of the wild-type (T) variant by random indels to allow only the protective form to be active is logical. Indeed, this will always depend on the availability of suitable PAM sites and the possibility of achieving allele specificity of the target SNP.

Single-base editing, with its ability to specifically replace one base pair with another, will always offer more accurate editing for transition SNPs; for example, for the protective rs641153 SNP (A), single-base editing of the risk (wild-type) (G) allele into an (A) seems to be an ideal strategy.

When single-base editing is not applicable, for example, for purine>pyrimidine transversion SNPs, as with rs2230199 (C>G) and rs4511667 (T>A) above, and no suitable PAM sites are available for active Cas9 editing, a prime editing approach can be harnessed to yield a successful edit.

Another study by Smailhodzic et al. further investigated the effect of the *CFB* protective haplotype rs4151667 on complement levels in a slightly larger cohort. The T>A change in rs4151667 reults in a Leucine to a Histamine replacement at amino acid 9 in the *CFB* locus (Leu9His), which resulted in a significant elevation in the activity of the alternative pathway in AMD patients compared with the controls, not the classic or lectin pathways. Carriers of the protective (His) allele presented with reduced FB levels, suggesting a lower complement activation in those individuals, which may explain the protective action of this haplotype [54]. The authors used a technique to measure C3 activation status in AMD called “C3d/C3 ratio”. Since it is a transversion SNP, a prime editing approach to turn the risk (Leu) allele into the protective (His) allele could potentially slow down the complement activation in those patients and lower the C3d/C3 ratio, leading to decreased activity of the alternative pathway.

Activation of the complement system is a dynamic process, and the complement landscape is constantly changing during the different phases of activation. During the acute phase, different complement components are rapidly generated, inactivated, and cleared. Haplotypes in different complement genes are associated with increased/decreased levels of different activation components. The C3d/C3 ratio is calculated by normalising the C3d over C3 plasma levels in AMD patients compared with the controls [54,55]. Several studies utilised the C3d/C3 ratio to investigate the effect of genetic variants on systemic complement activation. Work by den Hollander’s group suggested that the risk conferred by the common p.Tyr402His (rs1061170) in the *CFH* locus is associated with an elevated C3d/C3 ratio [54]. It is a C>T variant, where the (C) is the normal variant and the (T) is the pathogenic variant. As the most common SNP associated with AMD, CRISPR editing of the (T) pathogenic variant into the normal (C) variant is expected to result in a lower C3d/C3 ratio and decreased alternative pathway activity in affected individuals. However, this is currently not viable due to the available PAM sites not falling within an optimal seed window for editing, as discussed earlier in the manuscript. Several other AMD risk variants in the *CFH* locus are also reported to be associated with elevated complement activation. A study by Lores-Motta et al. looked at SNPs causing an elevated AMD risk, such as rs1410996 and rs3753396 in the *CFH* gene and rs6685931 in the *CFHR4* gene [56]. CRISPR manipulation of these SNPs may lead to a decreased C3d/C3 ratio and slow down the activity of the alternative pathway. The opposite effect could potentially result from manipulation of protective SNPs. The same study by Lores-Motta et al. identified the protective SNPs rs10922109 in the *CFH* locus and rs116503776 in the *C2/CFB* locus as being associated with a decreased C3d/C3 ratio [56]. CRISPR editing of the pathogenic SNPs into the protective variants could potentially result in decreased complement activity as measured by the C3d/C3 ratio. The same study also identified a risk variant in the CFI locus (rs141853578) as being associated with increased C3a/C3 activity. FI, as a complement inhibitor, is indeed a relevant target for CRISPR editing since rare risk variants are often associated with reduced FI levels. Activating FI may effectively re-establish its inhibitory effect on the alternative complement pathway.

CRISPR manipulation of AMD variants could potentially unveil some poorly understood mechanisms in complement activation. For example, conflicting results were reported in the literature on systemic FH levels, with some reports suggesting the involvement of FH in complement activation and some not [54,57,58,59,60,61]. A possible explanation could be attributed to different factors, such as variability in the size of cohorts and sensitivity/cross-reactivity of the assay used to measure complement activation, given how highly homologous FH is for the FHL proteins. For example, the largest, and most recent, study using FH-specific antibodies did not detect differences in the systemic levels of FH between carriers of AMD risk variants in *CFH* and controls [62]. However, they identified elevated FHR-4 levels in individuals with risk variants in the *CFH* locus. AMD-risk-conferring variants in the *CFH* locus, such as rs570618 and rs187328863, were found to be associated with increased levels of FHR-4, while protective variants (rs10922109 and rs61818925) were found to be associated with decreased FHR-4 levels. In addition, a previous study reported decreased FHR-1 levels in AMD patients [57], which is unexpected since deletions in *FHR-1* and *FHR-3* have been reported to have a protective effect against AMD [57]. In that study, samples from FHR-1-deficient-donors were positive for the FHR-1 protein, suggesting that the ELISA used to detect FHR-1 was not specific for FHR-1. The authors then explained that this was due to the cross-reactivity with FHR-2 and FHR-5, which are highly homologous with FHR-1. The discrepancies in the examples above could be explained by the homologous nature of the FH locus and its interacting FHR proteins. CRISPR manipulation of the different FHR proteins could potentially help in investigating the cross-reactivity of FH with FHR proteins, as knocking down one FHR can eliminate the possibility it is competing with FH for the binding of C3b and the subsequent activation of the pathway in the cascade. CRISPR-Cas systems allow manipulation with multiple gRNAs to achieve multiple iterations of knockdown or silencing. This is particularly important since FHR proteins are secreted systemically, not locally in the retina. CRIPSR manipulation of the different FHR proteins may help us in understanding the mechanisms of systemic versus local complement activation in AMD.

## 7. Conclusions and Remarks

AMD is a multifactorial disease with many factors influencing disease risk and progression. Although the complement system involvement in AMD is well established and progress has been made in understanding many of the mechanisms involved, the versatility of the complement system itself present a challenge to unveiling its functional implications in AMD. The debate on whether AMD is a local or systemic disease is ongoing [63], and it remains to be determined whether the altered complement levels in systemic circulation are reflection of the AMD variants, which exert their effect throughout the body, or if the effect is local to the retina and contributes to AMD manifestation. Currently, there is strong evidence of both local and systemic complement dysregulation contributing to AMD. In addition, the involvement of non-genetic factors in AMD’s pathogenicity, such as smoking and obesity, presents another challenge in isolating the main player in disease manifestation.

CRISPR/Cas systems, with their multiple pathways, including Cas9-induced DNA double-strand breaks, single-base editing, prime editing, and transcriptional regulation (activation/repression), present promising therapeutic avenues for the manipulation of disease variants. CRIPSR not only expands our knowledge on the molecular biology of disease mechanisms, which is still lacking in AMD; it opens a whole range of therapeutic applications, which fall beyond the focus of this review (several other reviews discuss its current applications [23,24,64,65,66,67,68,69]). In addition, the contribution of non-genetic factors in the pathogenesis of AMD, such as environmental factors, opens a wide range of considerations in implementing future therapeutic applications. The identification of many AMD risk variants in the complement system, and their involvement in disease incident and manifestation, raised many questions on whether it is therapeutically viable to manipulate such variants. CRISPR-Cas systems offer ideal platforms for the targeted editing of disease variants; however, their long-term success rate and efficacy remain unknown. Different CRISPR-Cas systems, and various Cas9 strains, can potentially be suitable for different variants depending on the nature of the nucleotide change and the availability of suitable PAM sites. One of the main challenges in targeting AMD risk variants in the complement system with CRISPR is achieving allele specificity of the disease variants. There are many factors that need to be considered: (i) the availability of suitable PAM sites, (ii) potent gRNAs, and (iii) choice of targeting approach. The availability of suitable PAM sites within a targetable range of the SNP is important as this will affect the targeting approach. Different Cas9 strains have different target ranges, so targeting efficiency and allele specificity will depend on whether the target variant falls within the target range of the chosen Cas9 strain. The editing approach, e.g., active Cas9 editing by indels, single-base editing, prime editing, or epigenetic editing, will largely depend on the proximity of the PAM sites that put the target variant within the seed window of the targeting approach. A lot of progress has been made in gRNA design, treatment, and screening techniques, which have facilitated fast and efficient editing of disease variants with CRISPR (for a recent review, see [70]). Numerous Cas9 variants with flexible PAM sites have been validated and show promising therapeutic potential (see [71] for more details). However, there are still hurdles that need to be overcome. Factors that affect the editing approach, such as the nature of the variant and whether it is targetable with a base-editing approach, still represent a challenge. Another consideration, which also depends on the proximity of PAM sites, is the efficiency of editing with CRISPR. Unlike editing in vitro, which is relatively easy due to the progress made with CRISPR editing mentioned above, the in vivo use of CRISPR/Cas systems faces many challenges, such as the recurrence of off-targeting effects, the unexpected/unpredictable immune response long term [72,73,74], and lastly, ethical concerns [75].

Although the availability of proximal PAM sites within a seeding window for active Cas9 editing suggests the editing of SNPs is viable, achieving allele-specific targeting is rather difficult. The fundamental studies on the specificity of the Cas9 enzymes show contrasting results for the number of bases that pose mismatch tolerance between the gRNA and the target DNA sequences (gRNA:DNA). The difficulty seems to be related to the position of the target SNP. It appears that mismatches in the distal region (5′ terminal) of the gRNA are more tolerable and do not result in DNA cleavage disruption, whereas mismatches in the PAM proximal region (3′ terminal of the gRNA) are less tolerable and will almost certainly result in disruption of cleavage by SpCas9. A study by Jinek et al. showed that up to six mismatches in the 5′ terminal of the gRNA did not disrupt DNA cleavage. However, a single mismatch in the 3′ terminal of the gRNA resulted in a disruption of DNA cleavage [76]. Cong et al. reported up to 9 bases in the 5′ terminal of the gRNA that are tolerable to gRNA:DNA mismatches, whereas a single mismatch within the first 11 bases of the 3′ terminal of the gRNA completely disrupt DNA cleavage [32]. Later, Hsu et al. described a varying degree of gRNA:DNA tolerance within all 20 bases of the gRNA [29]. The most tolerable gRNA:DNA mismatch, according to Doench et al., is what is known as rG:dT (where T appears in the target DNA with G instead of A in the complementary position in the gRNA) [77]. Thus, the use of the CRISPR-Cas system to distinguish between small differences in DNA sequences, such as those represented by SNPs, may pose a challenge for allele specificity due to this gRNA:DNA mismatch tolerance (reviewed in [78]).

Finally, the functional assessment of the CRISPR targeting of AMD risk variants will likely represent the biggest challenge due to the lack of robust and repeatable functional assays that can recognise different complement components. Future research should focus on addressing some of the challenges mentioned above regarding the molecular mechanisms of the complement system in AMD, explore novel methodologies in the functional assessment of AMD variants, and finally, further investigate targeting strategies for the CRISPR targeting of AMD variants.

## Figures and Tables

**Table 1:** A selection of AMD-related SNPs in complement factors *CFH*, *CFB*, *C3*, and *CFI* genes, showing targeting possibilities with different Cas9 strains.

SNP	Gene	SaCas9 PAM	SpCas9 PAM	xCas9 PAM	Nme2 Cas9 PAM	CJCas9 PAM	SNP (Intron/Exon)	Consequence
rs1410996 G>A	*CFH*	Y	Y	(NG)Y, (GAA)N, (GAT)N	Y	N	Intron	Pathogenic/risk factor
rs380390 G>A,C,T	*CFH*	N	Y	(NG)Y, (GAA)N, (GAT)N	Y	N	Intron	Pathogenic/intron variant
rs800292 G>A	*CFH*	N	Y	(NG)Y, (GAA)Y, (GAT)N	Y	N	Exon	Pathogenic/risk factor, missense variant
rs1061147 A>C	*CFH*	N	Y	(NG)Y, (GAA)Y, (GAT)N	N	Y	Exon	Pathogenic/synonymous variant
rs1831282 A>C	*CFH*	N	N	(NG)Y, (GAA)N, (GAT)Y	N	N	Intron	Pathogenic/intron variant
rs1061170 C>T	*CFH*	Y	N	(NG)Y, (GAA)N, (GAT)Y	Y	N	Exon	Pathogenic/risk factor/missense variant
rs1329424 T>G	*CFH*	N	N	(NG)Y, (GAA)Y, (GAT)N	Y	Y	Intron	Pathogenic/intron variant
rs1329428 C>T	*CFH*	N	N	(NG)Y, (GAA)Y, (GAT)N	N	N	Intron	Pathogenic/intron variant
rs10733086 A>C,T	*CFH*	N	N	(NG)Y, (GAA)N, (GAT)N	N	N	Intron	Pathogenic/intron variant
rs10737680 A>C	*CFH*	N	N	(NG)Y, (GAA)N, (GAT)N	Y	Y	Intron	Pathogenic/intron variant
rs10801555 A>G	*CFH*	N	N	(NG)Y, (GAA)Y, (GAT)N	Y	N	Intron	Pathogenic/intron variant
rs10922109 C>A	*CFH*	N	N	(NG)Y, (GAA)N, (GAT)Y	N	Y	Intron	Pathogenic/intron variant
rs35292876 C>T	*CFH*	N	Y	(NG)Y, (GAA)Y, (GAT)N	Y	Y	Exon	Benign/coding seq variant/Syn variant
rs121913059 C>T	*CFH*	N	N	(NG)Y, (GAA)Y, (GAT)N	N	N	Exon	Benign/missense variant
rs641153 G>A/T	*CFB*	Y	Y	(NG)Y, (GAA)N, (GAT)Y	Y	N	Exon	Protective
rs4541862 T>C	*CFB*	Y	Y	(NG)Y, (GAA)N, (GAT)Y	Y	Y	Exon	Pathogenic/intron variant
rs4151667 T>A	*CFB*	Y	Y	(NG)Y, (GAA)N, (GAT)N	Y	N	Exon	Benign/coding seq variant/missense
rs2230199 G>C,T	*C3*	N	Y	(NG)Y, (GAA)Y, (GAT)N	Y	N	Exon	Pathogenic/missense variant
rs147859257 T>G	*C3*	Y	Y	(NG)Y, (GAA)N, (GAT)N	Y	N	Exon	Pathogenic/missense variant
rs141853578 C>T	*CFI*	N	Y	(NG)Y, (GAA)Y, (GAT)N	Y	N	Exon	Pathogenic/missense variant
rs10033900 T>C	*CFI*	N	Y	(NG)Y, (GAA)Y, (GAT)N	Y	N	Intron	Pathogenic/intron variant

PAM: Protospace-adjacent Motif; SNP: Single Nucleotide Polymorphisms; SaCas9: *Staphylococcus aureus* Cas9; SpCas9: *Streptococcus pyogenes* Cas9; Nme2 Cas9: *N. meningitidis* Cas9; CJCas9: *Campylobacter jejuni* Cas9; Y: Yes; N: No.
